# Reticulocalbin 1 is required for proliferation and migration of non‐small cell lung cancer cells regulated by osteoblast‐conditioned medium

**DOI:** 10.1111/jcmm.17040

**Published:** 2021-11-07

**Authors:** Haijing Fu, Rui Chen, Yue Wang, Yang Xu, Chun Xia, Bing Zhang

**Affiliations:** ^1^ Cancer Research Center School of Medicine Xiamen University Fujian China; ^2^ Zhongshan Hospital Xiamen University Xiamen Fujian China

**Keywords:** ER stress, migration, NSCLC cell, osteoblast‐CM, proliferation, RCN1

## Abstract

Reticulocalbin1 (RCN1) is implicated in tumorigenesis and tumour progression. However, whether RCN1‐mediated bone metastasis of non‐small cell lung cancer (NSCLC) cells was elusive. Here, we assessed the effect of osteoblast‐conditioned medium (CM) on proliferation and migration of NSCLC cell line, NCI‐H1299 and NCI‐H460 cells, and identified the soluble mediators in CMs from osteoblasts and NSCLC cells using MTT, Clonogenicity, Transwell, wound healing, RT‐PCR, and Western blotting assays, and LC‐MS/MS analysis, respectively. Furthermore, the role of RCN1 was investigated in NSCLC cells cultured with or without osteoblast‐CM. Tumour growth and bone resorption were measured in a nude mouse model bearing NCI‐H1299 cells transduced with shRNA/RCN1 vector using *in vivo* imaging technique and micro‐CT. The results showed that RCN1 with a higher abundance in osteoblast‐CM, which was present in extracellular vesicles (EVs), enhanced RCN1 expression in NSCLC cells. Osteoblast‐CM partially offset the inhibitory effect of RCN1 depletion on proliferation and migration of NSCLC cells. RCN1 depletion‐induced endoplasmic reticulum (ER) stress caused by increasing GRP78, CHOP, IRE1α, p‐IRE1α, p‐PERK and p‐JNK, which was positively regulated by self‐induced autophagy, contributed to suppression of proliferation and migration in NCI‐H1299 cells. Therefore, osteoblasts produced RCN1 to transfer into NSCLC cells partially through EVs, facilitating proliferation and migration of NSCLC cells via blocking ER stress. RCN1 could be required for proliferation and migration of NSCLC cells regulated by osteoblast‐CM.

## 1 INTRODUCTION

Osteoblast is one of main cell types in bone microenvironment and has been reported to play key roles in cancer dissemination to bone and subsequent metastases.^1^, ^2^ Accumulating evidence shows that osteoblasts could produce factors into bone microenvironment, thereby affecting proliferation and metastases of cancer cells.^3^ Osteoblast‐derived sphingosine 1‐phosphate induces proliferation and confers resistance to therapeutics to bone metastasis‐derived prostate cancer cells.^4^ HIF signalling in osteoblast‐lineage cells promotes systemic breast cancer growth and metastasis in mice.^5^ Lung tumour‐associated osteoblast‐derived bone morphogenetic protein‐2 increased epithelial‐to‐mesenchymal transition of cancer by Runx2/Snail signalling pathway.^6^ Hence, advances in understanding the function and regulation mechanism of osteoblast‐derived factors in tumour growth and bone metastasis will aid in controlling and ultimately preventing cancer cell metastasis to bone.

Reticulocalbin1 (RCN1), a member of the CREC family, is an endoplasmic reticulum (ER)‐resident Ca2+‐binding protein.^7^ In addition to localizing in the ER lumen, RCN1 is also expressed on the cell surface, such as bone endothelia cell and prostate cancer cell, and its expression is heterogeneous in each constituent cell of some organs.^8^ RCN1 has been drawn intensive attention due to its role in tumorigenesis and tumour progression.^8^, ^9^, ^10^, ^11^, ^12^, ^13^ For example, RCN1 is expressed in the highly invasive breast cancer cell lines, not in poorly invasive ones.^9^ RCN1 appears as a potential renal cell carcinoma tumour marker in clinical proteomic analysis.^10^ RCN1 plays an important role in the development of doxorubicin‐resistant uterine cancer.^11^ Specially, in non‐small cell lung cancer (NSCLC), overexpression of RCN1 correlates with poor prognosis and progression^12^ and the efficacy of platinum‐based adjuvant chemotherapy relates with co‐expression of RCN1 and GST‐π.^13^ The involvement of RCN1 is thus required for lung cancer progression, although its regulatory mechanism is not yet clear.

Recent evidence displays that mRNA of RCN1 has been detected in extracellular vesicles (EVs) from porcine adipose tissue‐derived mesenchymal stem cells^14^ and osteoblasts.^15^ Compared to non‐sclerotic osteoblast secretome in osteoarthritis subchondral bone, RCN1 is one of 13 proteins significantly less secreted by sclerotic osteoblasts.^15^ Hence, there is a possibility that RCN1 is one of osteoblast‐derived factors to carry out its function in bone microenvironment. However, whether and how osteoblasts come in touch with proliferation and migration of NSCLC cells through producing RCN1 into bone microenvironment is not elucidated.

Here, we assessed the effect of osteoblast‐conditioned medium (CM) on proliferation and migration of non‐small cell lung cancer (NSCLC) cell line, NCI‐H1299 and NCI‐H460 cells, and identified the soluble mediators in CMs from osteoblasts. Furthermore, the role of RCN1 was investigated in NSCLC cells cultured with or without osteoblast‐CM. Our findings demonstrated that osteoblasts produced RCN1 to transfer into NSCLC cells partially through encapsulating in EVs, which facilitated proliferation and migration of NSCLC cells.

## 2 MATERIALS AND METHODS

### 2.1 Cell culture

NSCLC cell line, NCI‐H1299 and NCH‐H460 cells, and HEK293T cells were obtained from the Shanghai Institute of Cell Biology, Chinese Academy of Sciences (Shanghai, China). NCI‐H1299 and NCI‐H460 cells were maintained in RPMI‐1640 medium. HEK293T cells were cultured in DMEM with high glycose. After approved by the Ethics Committee of Medical School, Xiamen University, China (No.20170312) and the Committee on the Ethics of Animal Experiments of Xiamen University (No.20170196), human primary osteoblasts were isolated from the subchondral bone of patients undergoing total hip arthroplasty and cultured in DMEM/F12, and rat primary osteoblasts were isolated from the calvaria of neonatal Sprague‐Dawley rats (within 24–72 h after birth) and cultured in αMEM. The media (Hyclone, Logan, UT, USA) were supplemented with 10% foetal bovine serum,100 U/ml penicillin and 100 μg/ml streptomycin, and the cells were cultured at 37°C in a water‐saturated atmosphere of 5% CO2.

### 2.2 Osteoblast‐CM preparation

Human and rat primary osteoblasts were seeded at a density of 1x10^6^ in 60‐mm Petri dish and cultured in DMEM/F12 and α‐MEM for 48–72 h, respectively. The media were centrifuged to remove cells debris, and the supernatants were collected as osteoblast‐CM. According to subsequent experimental designs, NSCLC cells were cultivated in RPMI‐1640 or a 1:1 mixture of RPMI‐1640 and osteoblast‐CM.^4^

### 2.3 Cell proliferation

NSCLC cells were seeded in 96‐well plates and cultured in different types of medium for 48 h. Cell viability was measured by 3‐(4, 5‐Dimethylthiazol‐2‐y)‐2,5‐diphenyl‐tetrazolium bromide (MTT) assay at the end of the assay period.^16^, ^17^ Clonogenicity was also used to assess cell proliferation. Cells cultured in 6‐well plates for 48 h were fixed with 100% methanol and stained with 0.1% crystal violet, followed with observation of colonies under an Olympus BX41 microscope equipped with a digital camera (Olympus, Tokyo, Japan) at 4x magnification.^16^

### 2.4 Cell migration

Cell migration was assessed by a wound‐healing assay. Briefly, NSCLS cells were allowed to reach full confluence in 6‐well plates. A uniform scratch was made down the centre of the well using a 10 μl micropipette tip. After washed once with PBS, cells were cultured in different types of medium according to experimental designs. Images at 4× magnification were acquired at 48 h after scratching. 4 measurements were taken in each well, and the wound area was calculated using Image‐Pro Pus 6.0 system.^17^ Transwell migration assay was also used to assess cell migration. According to the previous studied,^16^, ^17^ cells in serum‐free RPMI‐1640 were placed into the top chambers of Transwell inserts set with 8 μm pore filters and different types of media were added to the bottom chamber, respectively. Cells on the top of the membrane were removed with a cotton swab. The chamber of Transwell was fixed with 4% paraformaldehyde and stained with 0.1% Giemsa stain. The migrated cells were observed under an Olympus BX41 microscope equipped with a digital camera (Olympus, Tokyo, Japan) at 4 × magnification.

### 2.5 MS sample preparation

Cells were cultured in opti‐MEM (Gibico) for 48 h. CM was collected and centrifuged at 3000 × *g* for 5 min to remove the dead cells and cell debris. The supernatants were concentrated to 1 ml in the filter unit (Amicon Ultra‐4, Millipore, MA, USA) and prepared for LC‐MS/MS analysis as described in previous studies.^18^, ^19^ Briefly, samples were reduced with 8 M urea(UA), 10 mm DTT and 50 mm iodoacetamide(IAA), digested with ice‐cold 0.01 μg/μL trypsin in 20 mm ammonium bicarbonate buffer(ABC), acidified with 50 μl 50% acetonitrile (ACN)/1% formic acid(FA) and desalted with 0.1% FA and C18. The samples were pooled, dried in a Speedvac (Ependorf, German) and resuspended with 0.1% FA. The concentration of samples was detected prior to LC‐MS/MS analysis.

### 2.6 LC‐MS/MS analysis

LC‐MS/MS analysis was carried out as previously described.^18^, ^19^ Briefly, combined with a nanoscale EASY‐nLC 1200UHPLC system (Thermo Fisher Scientific), Orbitrap Fusion Lumos equipped a nano‐electrospray source was used to perform all MS experiment. 0.1% FA and 80% MS‐grade ACN were utilized as Mobile phase A and B, respectively, both of which were dissolved in water. A nanoscale RP‐HPLC column (75 µm × 25 cm) packed with 2 µm C18 beads was used for the separation. The separation was maintained at a flow rate of 350 nl/min. The gradient applied ranging from 9% to 29% of Mobile phase B over 95 min and followed by a linear increase to 44% of Mobile phase B. For the MS/MS analysis, data‐dependent manner alternating between full‐scan MS and MS2 scans was operated to obtain datum. The spray voltage was set at 2.2 kV, and the temperature of the ion transfer tube was 300°C. Full scans were recorded in profile mode in the Orbitrap analyzer over a scan range from 350 to 1800 m/z with 120,000 resolutions. Target value for AGC was set to 4 × 105, and the maximal injection time was set to 50 ms. Selected ions were sequentially fragmented in a 3 seconds cycle by HCD with 30% normalized collision energy, specified isolated windows 1.6 m/z, 15,000 resolutions. AGC of 5 × 104 and 40 ms maximal injection time were used. Dynamic exclusion was set to 40 s. Unassigned ions or those with a charge of 2+ and >7+ were rejected for MS/MS.

### 2.7 MS data analysis

Proteome Discoverer (PD, version 2.2) was carried out to process raw data, and the reviewed SwissProt human proteome database (20259 entries) was used to data search. All searches were carried out with precursor mass tolerance of 20 ppm, fragment mass tolerance of 0.02 Da and three trypsin missed cleavages allowed. Only peptides with at least six amino acids in length were considered. The peptide and protein identifications were filtered by PD to control the false discovery rate (FDR) <1%. At least one unique peptide was required for protein identification.

Further annotation, visualization and integrated discovery for gene ontology analysis were performed by Metascape online service. Through Metascape, proteins with gene symbols were grouped by biological process. Grouped protein were calculated and visualized by Draw Venn Diagrams serve and InteractiVenn tools.^20^, ^21^

### 2.8 Extraction of osteoblast‐derived EVs

As described previously,^22^ reaching 80% confluency, osteoblasts were rinsed with PBS and cultured un serum‐free medium for 48 h. Osteoblast‐CM was collected and centrifuged at 300 × *g* for 15 min, 2000 × *g* for 15 min, and 10,000 × *g* for 30 min at 4°C to remove dead cells and cell debris. The supernatants were ultra‐ centrifuged at 120,000 x g for 3 h to separate EVs from CM, followed with the identification of EVs performed by western blotting assay.

### 2.9 Western blotting assay

Protein extracts were subjected to SDS‐PAGE (8–15%) and transferred to a PVDF membrane (GE Healthcare, Hertfordshire, UK) as described previously.^23^, ^24^ The membrane was incubated with various primary antibodies as required at 4°C overnight, followed by the addition of the corresponding secondary antibodies at room temperature for 1 to 2 h (Table 1). An enhanced chemiluminescence (ECL) detection kit was used to detect antibody reactivity (Pierce, Rockford, IL, USA).

**TABLE 1:** Information of antibodies

Antibody	Manufacturer and Number	Dilution con.	Time
Bcl−2	Abcam ab59348	1:500	4℃ overnight
PCNA	Proteintech 60097–1‐Ig	1:1000	4℃ overnight
JNK	Proteintech 66210–1‐Ig	1:1000	4℃ overnight
p‐JNK(phosphoT183+T183+T221)	Abcam ab124956	1:1000	4℃ overnight
ATG5	Abcam ab217179	1:1000	4℃ overnight
P62	Cell Signaling technology #5114	1:1000	4℃ overnight
Beclin1	Cell Signaling technology #2983	1:1000	4℃ overnight
PERK	Cell Signaling technology #3192	1:1000	4℃ overnight
p‐PERK(phosphoT980)	Bioss Bs−3330R	1:1000	4℃ overnight
IRE1α	Abcam ab37073	1:1000	4℃ overnight
p‐IRE1α (phosphoS724)	Abcam ab48187	1:1000	4℃ overnight
CHOP	Proteintech 15204–1‐AP	1:1000	4℃ overnight
GRP78	Abcam ab21685	1:1000	4℃ overnight
CD9	Proteintech 60232–1‐Ig	1:1000	4℃ overnight
CD63	Proteintech 25682‐−1‐AP	1:1000	4℃ overnight
CD81	Abcam ab21685	1:1000	4℃ overnight
c‐PARP	Abcam ab32064	1:1000	4℃ overnight
Bid	Santa Crus sc−373939	1:200	4℃ overnight
RCN1	Abcam ab184441	1:1000	4℃ overnight
mTOR	Cell Signaling technology #2983	1:1000	4℃ overnight
p‐mTOR(phosphoS2448)	Cell Signaling technology #2971	1:1000	4℃ overnight
ULK1	Abcam ab128859	1:1000	4℃ overnight
p‐ULK1(phosphoS757)	Cell Signaling technology #6888	1:1000	4℃ overnight
MMP2	Proteintech 66366‐−1‐Ig	1:1000	4℃ overnight
β‐actin	Sigma A3854	1:50000	RT 20 min
anti‐Mouse‐IgG(H+L)HRP	Proteintech SA00001‐1	1:25000	RT 1 h
anti‐Rabbit‐IgG(H+L)HRP	Proteintech SA00001‐2	1:25000	RT 1 h

### 2.10 Plasmid construction and transfection

Short hairpin RNA (shRNA) targeting RCN1 (shRCN1) was purchased from Gene Chem (Shanghai, China). ShRNA sequences were shown below,shRCN1‐1: 5’CcggagAAGCTAACTAAAGAGGAAACTCGAG TTTCCTCTTTAGTTAGCTTCTTTTTTg3’, shRCN1‐2: 5’ CcgggaCGGGAAGTTAGACAAAGATCTCGAG ATCTTTGTCTAACTTCCCGTCTTTTTg3’, shRCN1‐3: 5’CcggcaTCTTTGATAATGTCGCCAACTCGAG TTGGCGACATTATCAAAGATGTTTTTg3’. After cells were transduced with different shRCN1 vectors by a lentiviral transfection strategy, stable cell lines were obtained under the pressure of purpmycin (2 μg/ml, BioVision, Inc.). Additionally, cells were transiently transfected for 24 h with pHBLV‐CMV‐ZsGreen‐RCN1 vector expressing RCN1 (h‐RCN1) (HANBIO, Shanghai, China) using Lipofectamine 3000 (Invitrogen) according to the manufacturer's procedure and previous studies.^17^, ^25^

#### 2.10.1 Real‐time PCR (RT‐PCR) assay

Total RNA in different cancer cells was extracted using Trizol (Invitrogen, CA, USA) and then subjected to reverse transcription using a Primescript RT Master Mix Kit (Takara, Dalian, China) to synthesize cDNA. Real‐time PCR was then performed using a Light Cycler (Roche) with a SYBR Premix Ex Taq II Kit (Takara, Dalian, China). Results were analysed as described in previous studies.^25^, ^26^ The primers used for quantitative PCR are described in Table 2.

**TABLE 2:** Primers’ sequences of quantitative real‐time PCR

Gene name	Primer sequences
MMP2	Forward 5’‐AGTAAACAGCAAGAGAACCT‐3’ Reverse5’‐AACAGATGCCACAATAAAGC‐3’
RCN1	Forward 5’‐GGAAACCCTGGAGGACATCG‐3’ Reverse5’‐CCCGTCCTTGTTCAGATCCC‐3’
GAPDH	Forward 5’‐ TGCACCACCAACTGCTTAGC‐3’ Reverse5’‐ GGCATGGACTGTGGTCATGAG‐3’

### 2.11 *In vivo* animal model of bone resorption

All animal studies were conducted according to the regulations of the Institutional Animal Care and Use Committee protocol. This study was approved by the Committee on the Ethics of Animal Experiments of Xiamen University (No. 20170196). Twelve 6‐week‐old female BALB/Cnu/nu nude mice were purchased from Shanghai Slac Laboratory Animal Co. Ltd. (Shanghai, China) and randomly assigned to 2 groups (control (shcon) and shRCN1‐2, 6 mice per group). 100 μL stable NCI‐H1299 cells (2 × 10^6^/mouse) containing the shcon or shRCN1 vector were injected into the tibial medullary cavity of mouse two hind legs with a 1 ml syringe. Mouse body weight was measured every 5 days. Mice were sacrificed after 55 days and subjected to measurement of tumour growth with the IVIS Lumin II in vivo imaging (PerkinElmer) and bone resorption with the Skyscan 1272 micro‐CT imaging system (Bruker, Belgium).^27^, ^28^

### 2.12 Micro‐computed tomography

Mouse legs were fixed with 4% paraformaldehyde for 48 h prior to micro‐CT analysis as described previously.^29^, ^30^, ^31^, ^32^ It was examined that the qualitative and semi‐quantitative analysis of bone destruction in the two groups was measured using high‐resolution micro‐CT scanning (Skyscan 1272). Briefly, scanning was carried out at an X‐ray source voltage of 90 kV and beam current of 111 mA using 0.25 mm Al filter to capture the best X‐ray projections of mineralized tissue. The X‐ray projections were captured at rotation interval of 0.4°C around 360°C of the sample with 6 μm pixel resolution. The defect region was selected in the dataset for calibration using CTAn software. Various parameters were measured at thresholding of 0–255. The representative 3D images and mineralization mapping of each group were carried out using SkyScan CTVox software.^32^

### 2.13 Statistical analysis

Differences between the groups were examined for statistical significance using t test (between two groups) and one‐way ANOVA (among three groups or more) with GraphPad Prism 6 software (GraphPad Software). A value of *p* < 0.05 was considered significant.

## 3 RESULTS

### 3.1 Osteoblast‐CM facilitated proliferation and migration in NSCLC cells

**FIGURE 1:**
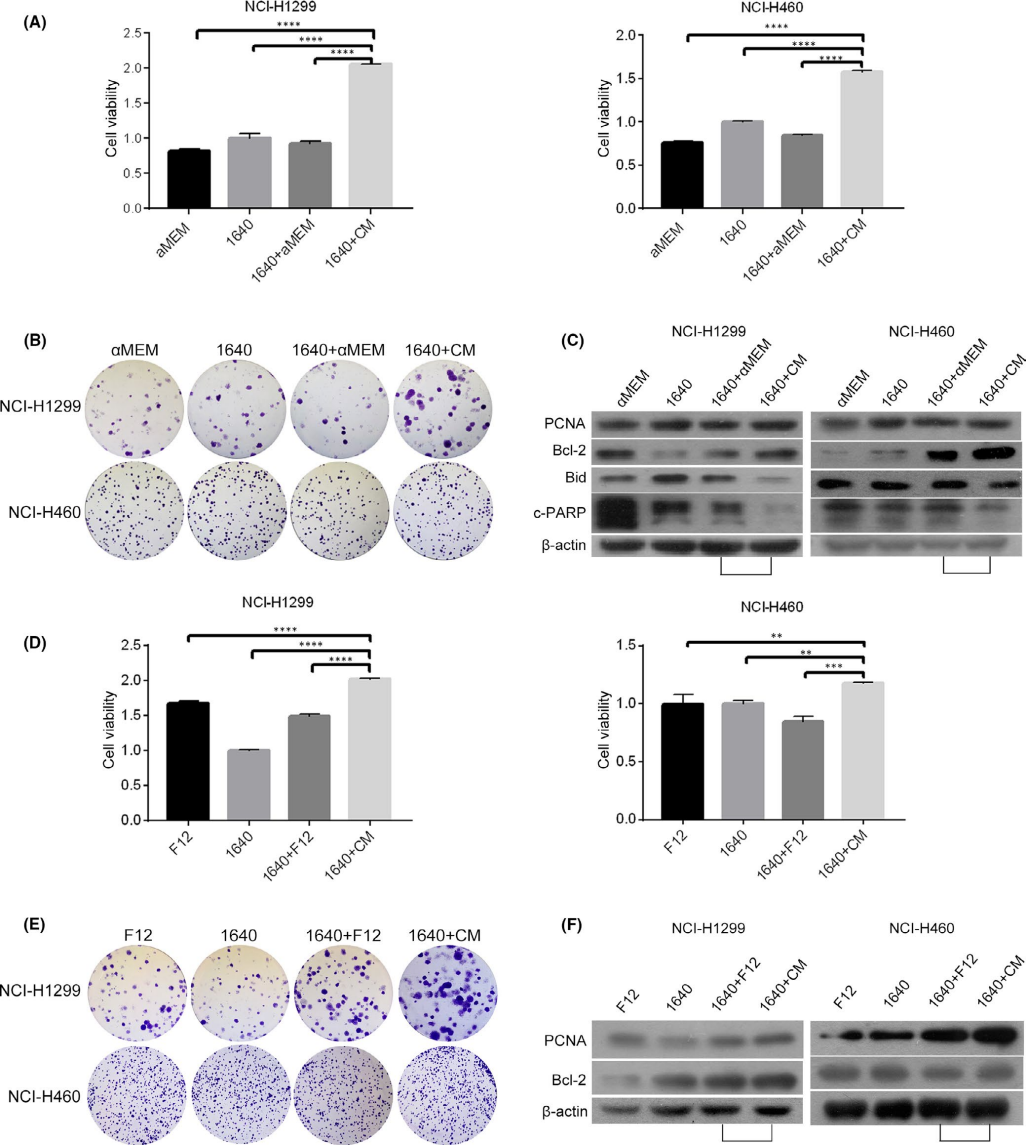
Conditioned medium (CM) from human or rat primary osteoblasts promoted proliferation in human lung cancer NCI‐H1299 and NCI‐H460 cells. A‐C, NCI‐H1299 and NCI‐H460 cells were cultured in αMEM, 1640,1640+αMEM (1:1), 1640+CM (1:1) from rat osteoblasts for 48 h, respectively. The cell viabilities were detected via MTT assay as described in the Material and methods section (A). The cell growth was detected via colony forming as described in the Material and methods section (B). The PCNA, Bcl‐2, Bid, c‐PARP and β‐actin protein levels were detected via Western blotting as described in the Material and methods section (C). D‐F, NCI‐H1299 and NCI‐H460 cells were cultured in F12, 1640,1640+F12(1:1), 1640+CM (1:1) from human osteoblasts for 48 h, respectively. The cell viabilities were detected via MTT assay as described in the Material and methods section (D). The cell growth was detected via colony forming as described in the Material and methods section (E). The PCNA, Bcl‐2 and β‐actin protein levels were detected via Western blotting as described in the Material and methods section (F). The data are representative of three independent experiments (**p<0.01, ***p<0.001, ****p<0.0001)

**FIGURE 2:**
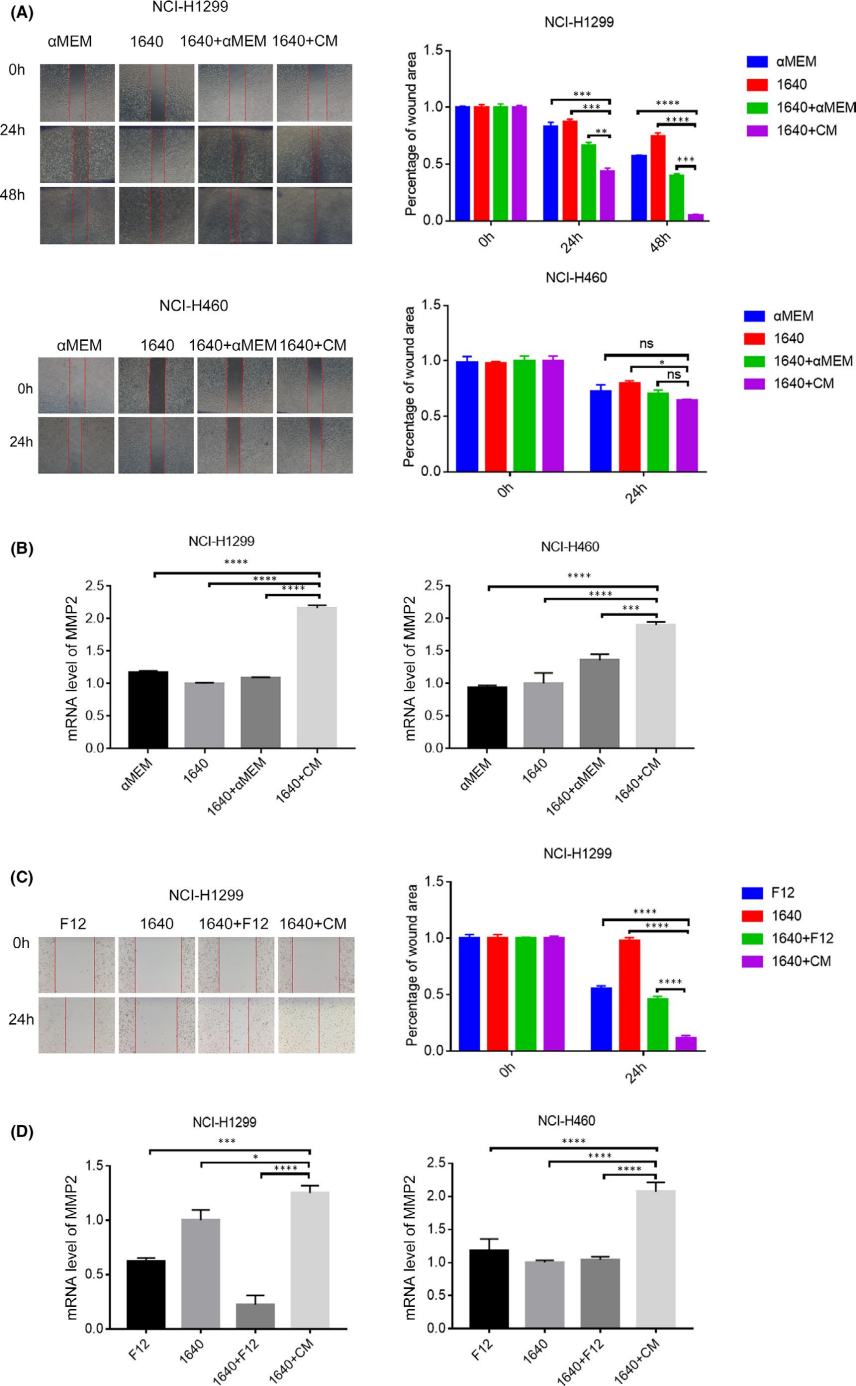
Conditioned medium (CM) from human or rat primary osteoblasts promoted migration in human lung cancer NCI‐H1299 and NCI‐H460 cells. A&B, NCI‐H1299 and NCI‐H460 cells were cultured in αMEM, 1640,1640+αMEM (1:1), 1640+CM (1:1) from rat osteoblasts for 48 h, respectively. As described in the Material and methods section, the cell migration was assessed using a wound healing assay (A). Relative mRNA level of MMP2 was measured via RT‐PCR as described in the Material and methods section (B). C, NCI‐H1299 cells were cultured in F12, 1640,1640+F12(1:1), 1640+CM (1:1) from human osteoblasts for 48 h, the cell migration was assessed using a wound healing assay as described in the Material and methods section. D, NCI‐H1299 and NCI‐H460 cells were culture in F12, 1640,1640+F12(1:1), 1640+CM (1:1) from human osteoblasts for 48 h, relative mRNA level of MMP2 was measured via RT‐PCR as described in the Material and methods section. The data are representative of three independent experiments (*p<0.05, **p<0.01, ***p<0.001, ****p<0.0001)

To determine the effect of osteoblast‐CM on proliferation and migration of NSCLC cells, NCI‐H1299 and NCI‐H460 cells were cultured with different types of media, respectively. As shown in Figure 1A, B, mixed medium (1640+CM,1:1 mixture of RPMI‐1640 and CM from rat primary osteoblasts) increased cell viability and colony formation (*vs* 1640+αMEM). Meanwhile, the protein level of PCNA and BCL‐2 increased accompanied with decreased Bid and cleavage‐PARP(c‐PARP) levels (Figure 1C). Similar results were observed in NCI‐H1299 and NCI‐H460 cells cultured with mixed medium (1640+CM, 1:1 mixture of RPMI‐1640 and CM from human primary osteoblasts) (Figure 1D–F, *vs* 1640+F12). In addition to promoting proliferation, 1640+CM (from rat osteoblasts) significantly reduced the percentage of wound area, accompanied with a significant increase of MMP2 at the mRNA level in NCI‐H1299 cells (Figure 2A, B, *vs* 1640+αMEM). 1640+CM caused a significant increase of MMP2 at the mRNA level in NCI‐H460 cells, although there was no significant difference in the percentage of wound area (Figure 2A, B, *vs* 1640+αMEM). Meanwhile, 1640+CM (from human osteoblasts) reduced the percentage of wound area in NCI‐H1299 cells (Figure 2C, *vs* 1640+F12). 1640+CM caused a significant increase of MMP2 at mRNA level in NCI‐H1299 and NCI‐H460 cells (Figure 2D, *vs* 1640+F12). As a result, osteoblast‐CM facilitated proliferation and migration of NSCLC cells.

### 3.2 RCN1 with a higher abundance in osteoblast‐CM enhanced RCN1 expression in NCI‐H1299 and NCI‐H460 cells

**FIGURE 3:**
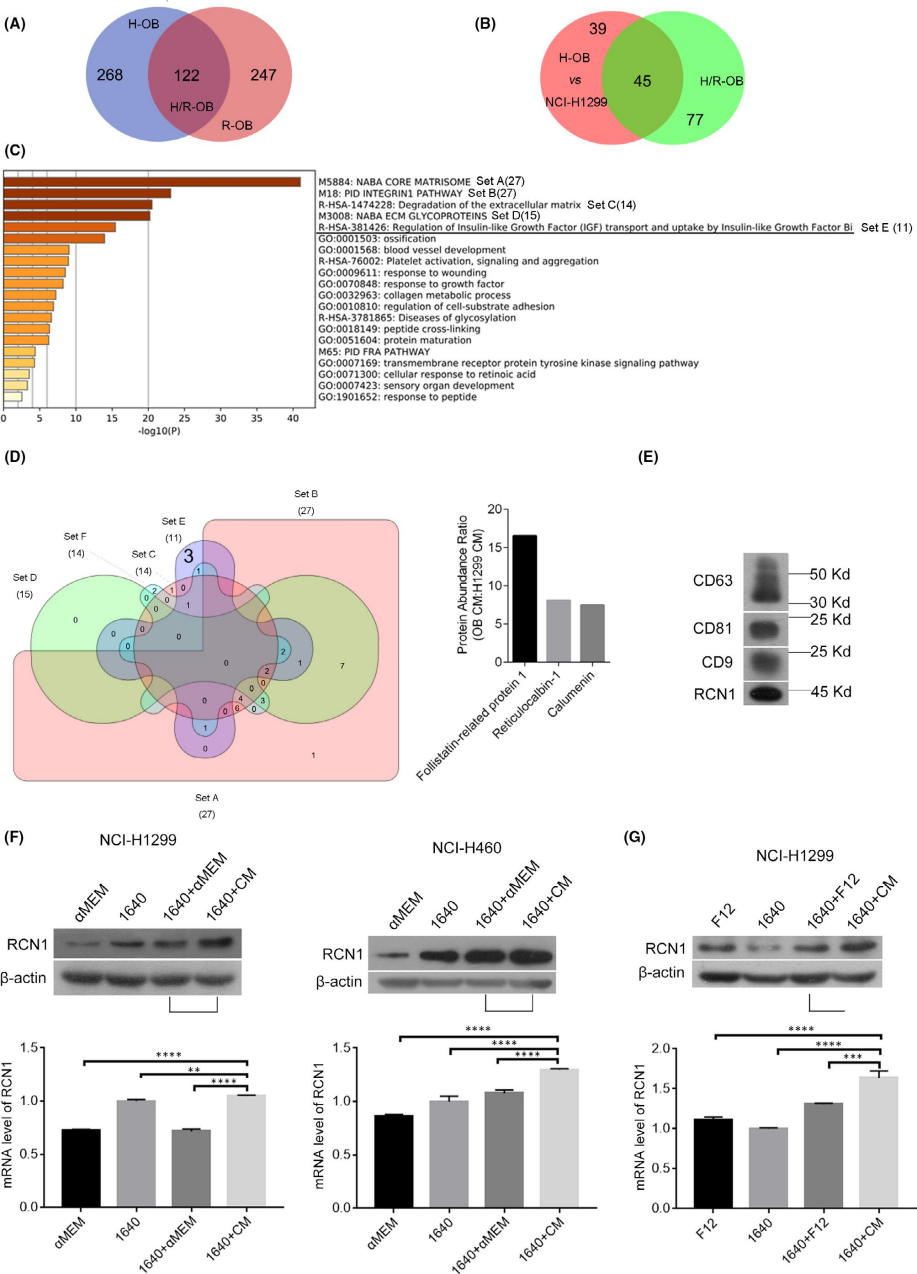
RCN1 with a higher abundance in osteoblast‐CM enhanced RCN1 expression in NSCLC cells. A‐D, As described in the Material and methods section, the soluble mediators in human or rat osteoblast‐CM and NCI‐H1299 cell‐CM were identified and analysed using LC‐MS/MS, prior to analysis by Metascape online service and calculation and visualization by Draw Venn by Diagrams serve and InteractiVenn tools. E, Using a gradient centrifugation as described in the Material and methods section, osteoblast‐derived exosomes were extracted and the CD9, CD63, CD81 and RCN1 protein levels were detected via Western blotting as described in the Material and methods section. F, NCI‐H1299 and NCI‐H460 cells were cultured in αMEM, 1640,1640+αMEM(1:1), 1640+CM(1:1) from rat osteoblasts for 48 h, the RCN1 and β‐actin protein levels and mRNA level of RCN1 were measured via Western blotting and RT‐PCR, respectively, as described in the Material and methods section. G, NCI‐H1299 cells were culture in F12, 1640,1640+F12(1:1), 1640+CM (1:1) from human osteoblasts for 48 h, the RCN1 and β‐actin protein levels and mRNA level of RCN1 were measured via Western blotting and RT‐PCR, respectively, as described in the Material and methods section. The data are representative of three independent experiments (**p<0.01, ***p<0.001, ****p<0.0001)

To confirm the crucial elements in osteoblast‐CM regulating cell proliferation and migration of NSCLC cells, the soluble mediators in human or rat osteoblast‐CM and NCI‐H1299 cell‐CM were identified and analysed using LC‐MS/MS. As described in Figure 3A, between human and rat osteoblast‐CM (H‐OB and R‐OB), from the top 40% high‐abundance proteins, 122 types of proteins were shared. Furthermore, between human osteoblast‐CM and NCI‐H1299 cell‐CM (H‐OB and HCI‐H1299), there were 84 types of shared proteins in the top 10% differentially expressed proteins that were expressed at relatively higher levels in osteoblast‐CM and lower levels in NCI‐H1299‐CM, and 45 types of shared proteins were then identified in the above mentioned 122 and 84 types of proteins using Metascape (Figure 3B). Excepting from those proteins regulating extracellular matrix of osteoblasts, we were interested in the group of regulation of insulin‐like growth factor transport and uptake by insulin‐like growth factor Bi was identified, which contained RCN1(Reticulocalbin1), CALU(Calumenin) and FSTL1 (Follistatin‐like 1) (Figure 3C, D. Among them, RCN1 was particularly interested as overexpressed RCN1 is correlated with poor prognosis and progression in lung cancer^12^ (Figure S1). Subsequently, the released RCN1 was detected in EVs from rat osteoblast‐CM using gradient centrifugation (Figure 3E). To verify whether RCN1 with a higher abundance in osteoblast‐CM might have an influence on RCN1 expression in NSCLC cells, NCI‐H1299 or NCI‐H460 cells were cultured in mixed medium (1640+CM, 1:1 mixture of RPMI‐1640 and CM from rat or human osteoblasts) and the protein and mRNA expression levels of RCN1 were measured using RT‐PCR and Western blotting assays, respectively. The results showed that rat osteoblast‐CM increased the mRNA and protein levels of RCN1 in NCI‐H1299 and NCI‐H460 cells (Figure 3F, vs 1640+αMEM), as well as human osteoblast‐CM in NCI‐H1299 cells (Figure 3G, vs 1640+F12). Therefore, RCN1 with a higher abundance in osteoblast‐CM enhanced RCN1 expression in NCI‐H1299 and NCI‐H460 cells partially via encapsulating in EVs.

### 3.3 Osteoblast‐CM partially offset the inhibitory effect of RCN1 depletion on proliferation and migration in NCI‐H1299 cells

**FIGURE 4:**
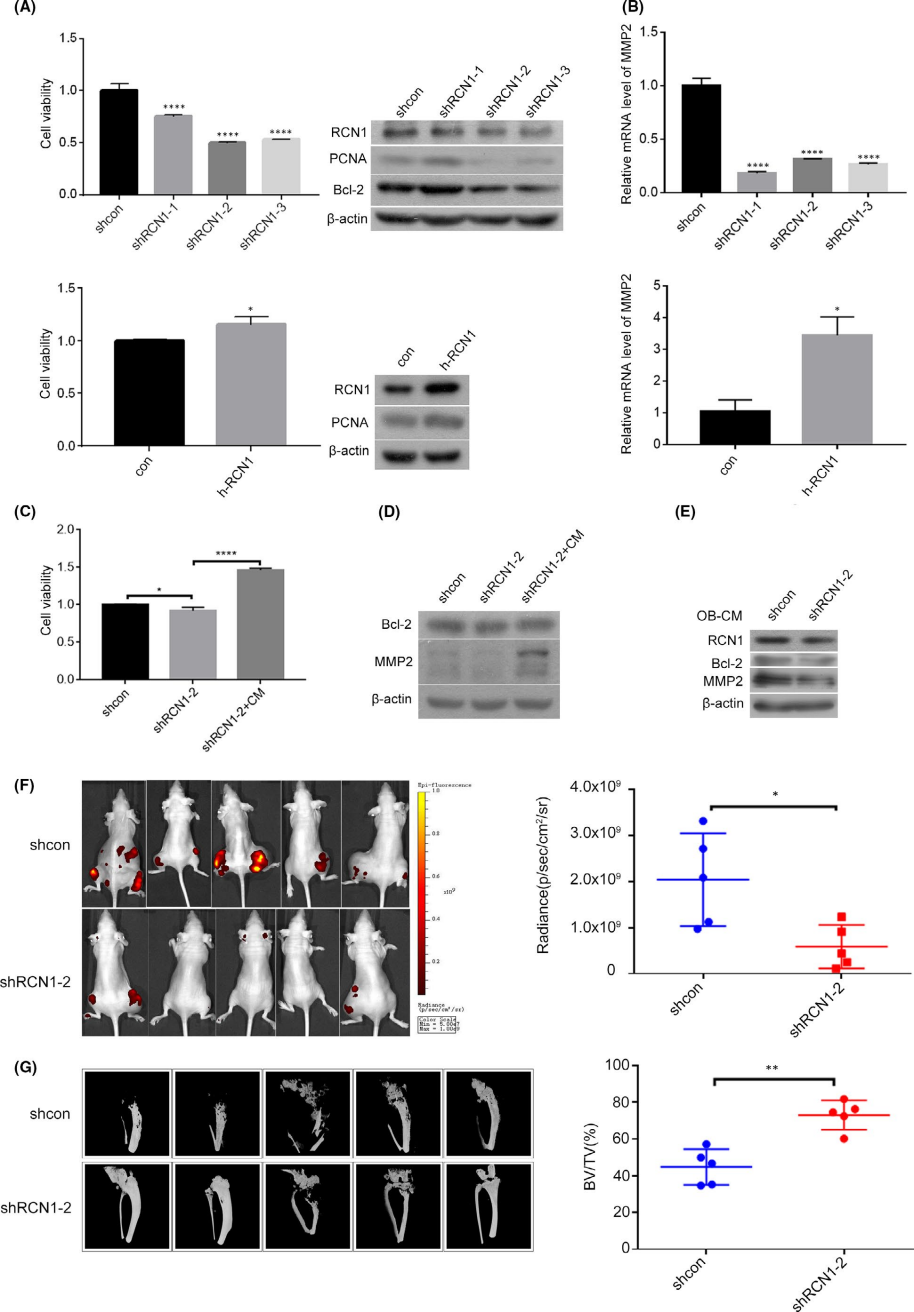
Osteoblast‐CM offset partly the inhibitory effect of RCN1 depletion on cell proliferation and migration in NCI‐H1299 cells. A&B, NCI‐H1299 cells were stably transduced with shRNA/RCN1‐1/2/3 or transiently transfected with GFP‐RCN1 vectors. The cell viabilities were detected via MTT assay and the PCNA, RCN1, Bcl‐2 and β‐actin protein levels were measured via Western blotting as described in the Material and methods section (A). Relative mRNA level of MMP2 was measured via RT‐PCR as described in the Material and methods section (B).C&D, NCI‐H1299 cells were stably transduced with shRNA/RCN1‐2 vector and cultured in rat osteoblast‐CM. The cell viabilities were detected via MTT assay(C) and the MMP2, Bcl‐2, and β‐actin protein levels (D) were measured via Western blotting as described in the Material and methods section. E, NCI‐H1299 cells were cultured in CM from rat osteoblasts transduced with shRNA/RCN1‐2 vector. The RCN1, Bcl‐2, MMP2 and β‐actin protein levels were measured via Western blotting. F&G, NCI‐H1299 cells transduced with shRNA/RCN1‐2 vector were injected in the proximal metaphysic of the tibia in nude mice for 8 weeks. The radiance (p/sec/cm2/sr) of tumour mass was measured using the IVIS Lumin II in vivo imaging (F). The BV/TV in cortical bone in the tibia was measured using high resolution micro‐CT scanning(G). The data are representative of three independent experiments (*p<0.05, **p<0.01, ****p<0.0001)

As shown in Figure 4A, RCN1 depletion using lentiviral‐mediated shRNA/RCN1‐1/2/3 vectors(shRCN1) caused a decrease of cell viability and the PCNA and Bcl‐2 levels, whereas the transfection with pHBLV‐CMV‐ZsGreen‐RCN1 vector expressing RCN1(h‐RCN1) had an opposite influence on them. Meanwhile, RCN1 depletion caused decreased MMP2 mRNA level and the number of migrated cells, whereas the transfection with h‐RCN1 vector rescued the effect of shRCN1 (Figure 4B; Figure S2). Nevertheless, the inhibitory effect of RCN1 depletion on cell viability and BCL‐2 and MMP2 protein expression were partially offset by the addition of human osteoblast‐CM (Figure 4C, D). CM from rat osteoblasts transduced with shRCN1‐2 vector led to decreased MMP2 and Bcl‐2 levels, accompanied with decreased RCN‐1 level in NCI‐H1299 cells (Figure 4E). Meanwhile, the mRNA level of MMP2 significantly decreased in NCI‐H1299 cells (Figure S3). Additionally, after NCI‐H1299 cells transduced with shRCN1‐2 vector were injected in the proximal metaphysic of the tibia in nude mice for 8 weeks, the radiance (p/sec/cm2/sr) of tumour mass in shRCN1‐2 group was significantly lower than that in shcon group(Figure 4F) and the BV/TV in cortical bone in the tibia in shRCN1‐2 group was significantly higher than that in shcon group (Figure 4G), representing that RCN1 depletion in NCI‐H1299 cells reduced tumour growth accompanied with decreased bone resorption in cortical bone. Therefore, osteoblast‐CM partially offset the inhibitory effect of RCN1 depletion on proliferation and migration in NCI‐H1299 cells.

### 3.4 RCN1 depletion‐induced ER stress contributed to suppression of proliferation and migration in NCI‐H1299 cells

**FIGURE 5:**
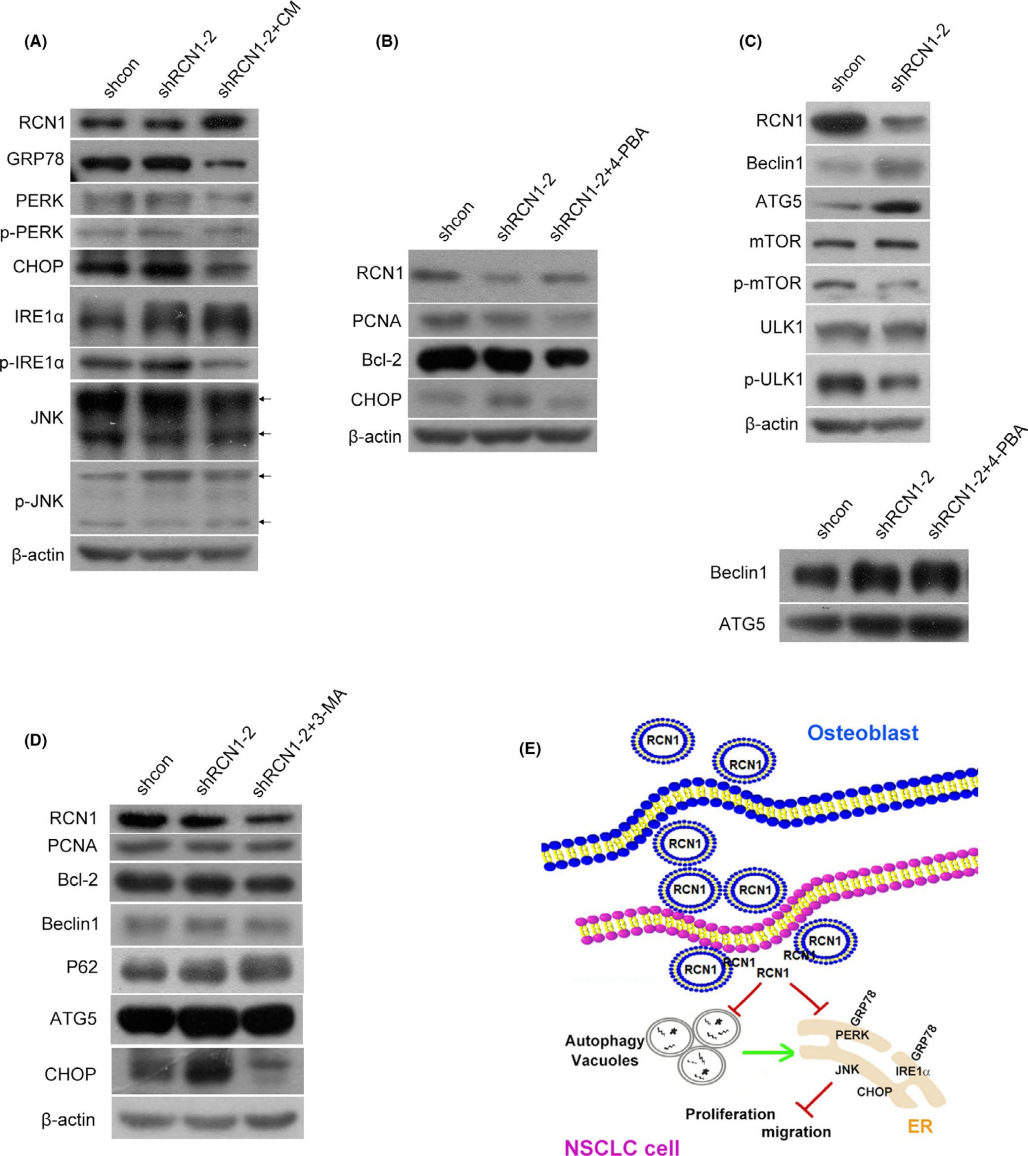
RCN1 depletion‐induced endoplasmic reticulum (ER) stress contributed to suppression of proliferation and migration in NCI‐H1299 cells. A, NCI‐H1299 cells stably transduced with shRNA/RCN1 vector were cultured in RIPA‐1640 and human osteoblast‐CM for 48 h, respectively. The GRP78, PERK, p‐PERK, CHOP, IRE1α,p‐IRE1α, JNK, p‐JNK, RCN1 and β‐actin protein levels were then detected via western blotting. B, NCI‐H1299 cells transduced with shRNA/RCN1‐2 vector were treated with 4‐PBA (1 mm) for 48 h and the RCN1, PCNA, Bcl‐2, CHOP and β‐actin protein levels were detected via Western blotting. C, NCI‐H1299 cells were transduced with shRNA/RCN1‐2 vector and the RCN1, Beclin1, ATG5, mTOR, p‐mTOR, ULK1, p‐ULK1 and β‐actin protein levels were detected via Western blotting (Upper panel). NCI‐H1299 cells transduced with shRNA/RCN1 vector were treated with 4‐PBA (1 mm) for 48 h, and the Beclin1 and ATG5 protein levels were detected via Western blotting (Lower panel). D, NCI‐H1299 cells transduced with shRNA/RCN1 vector were treated with 3MA (6 mm) for 48 h, and the RCN1, PCNA, Bcl‐2, Beclin1, P62, ATG5, CHOP and β‐actin protein levels were detected via Western blotting. The data are representative of three independent experiments. E, Schematic diagram illustrating the regulatory mechanism of osteoblastic RCN1 in NSCLC cells

Tumour microenvironment stresses could produce ER stress, which has been highlighted to the involvement in the development and progression of lung cancer.^33^, ^34^ To confirm whether the regulation of cell proliferation and migration by RCN1 was associated with ER stress in NSCLC cells, we investigated the protein expression levels of some ER stress‐related biomarkers. The results in Figure 5A showed that RCN1 depletion by shRCN1‐2 vector enhanced GRP78, p‐PERK(Thr980), CHOP, IRE1α, p‐IRE1α(Ser724) and p‐JNK(Thr183/Thr183/Thr221) levels without the alteration of total PERK and JNK levels, compared with shcon group. Nevertheless, the effect of RCN1 depletion by shRCN1‐2 vector on ER stress was neutralized by human osteoblast‐CM, even offset (Figure 5A). Given that ER stress can induce apoptosis and autophagy,^33^, ^35^ the expression levels of apoptosis‐ and autophagy‐related biomarkers were simultaneously detected in NCI‐H1299 cells transduced with shRCN1‐2 vector with or without ER stress inhibitor 4‐PBA. The results shown in Figure 5B exhibited that PCNA and Bcl‐2 levels were reduced by shRCN1‐2 vector, accompanied with an increase in CHOP level. The addition of ER stress inhibitor 4‐PBA led to decreased PCNA and Bcl‐2 levels compared with shRCN1‐2 group (Figure 5B). On the contrary, the depletion of RCN1 by shRCN1‐2 vector result in increased Beclin1 and ATG5 levels, accompanied with decreased p‐mTOR and p‐ULK1 levels in NCI‐H1299 cells (Upper panel in Figure 5C). However, compared with shRCN1‐2 group, the addition of ER stress inhibitor 4‐PBA only led to a slight increase in the ATG5 and Beclin1 levels (Lower panel, Figure 5C). To further confirm the relationship between ER stress, autophagy and apoptosis, autophagy inhibitor 3‐MA was added in NCI‐H1299 cells transduced with shRCN1‐2 vector. Compared with shRCN1‐2 group, 3‐MA addition caused a dramatic decrease in the CHOP level with a slight decrease in the Bcl‐2 level without an alteration of PCNA level (Figure 5D). Besides, an observation under transmission electron microscopy showed that the expansion of ER caused by the depletion of RCN1 with shRCN1‐2 vector was reduced by the addition of autophagy inhibitor 3MA, like ER stress inhibitor 4‐PBA (Figure S4). Collectively, RCN1 depletion‐induced ER stress might contribute to the reduction of proliferation and migration in NCI‐H1299 cells.

## 4 DISCUSSION

Previous studies have demonstrated that osteoblast‐CM was a potent chemoattractant for bone metastasis of cancer cells.^4^, ^6^, ^36^, ^37^ For example, factors derived from osteoblasts induce an expression signature that identifies prostate cancer metastasis.^36^ Bone metastatic breast cancer cells utilize osteoblast‐derived cytokines to facilitate breast cancer cell colonization and survival upon arrival in the bone microenvironment.^37^ Herein, we found that RCN1, which correlates with poor prognosis and progression of NSCLC, was identified to express with a higher abundance in human or rat primary osteoblast‐CM as well as in EVs. Subsequently, osteoblast‐CM significantly enhanced RCN1 expression, accompanied with increased proliferation and migration in NCI‐H1299 and NCI‐H460 cells, whereas CM from osteoblasts transduced with shRCN1 vector caused decreased MMP2 and Bcl‐2 levels in NCI‐H1299 cells. Based on the positive and negative data, it is suggested that osteoblasts produced RCN1 into bone microenvironment and in turn transferred into NSCLC cells partially through encapsulating in EVs, leading to an increase in the RCN1 expression level in NSCLC cells, which facilitated proliferation and migration of NSCLC cells. That is, like other osteoblast‐derived factors, such as sphingosine 1‐phosphate,^4^ HIF^5^ and BMP2,^6^ RCN1 might be another osteoblast‐derived factor to promote proliferation and migration of NSCLC cells. The other experimental data provided further supportive evidence for this notion, including that osteoblast‐CM offset the inhibitory effect of RCN1 depletion by shRCN1 vector on proliferation and migration in NCI‐H1299 cells and that bone resorption was suppressed in a nude mouse model bearing NCI‐H1299 cells transduced with shRCN1 vector.

RCN1 has been implicated in ER stress.^38^, ^39^ RCN1 suppresses ER stress‐induced apoptosis via calcium homeostasis and PERK–CHOP signalling in cancer cells.^38^ EGFRvIII promotes cell survival during ER stress through a RCN1‐dependent mechanism in glioblastoma cells.^39^ Herein, it was observed that canonical ER stress biomarkers,^33^ such as GRP78, p‐PERK(Thr980), CHOP, IRE1α, p‐IRE1α(Ser724) and p‐JNK (Thr183/Thr183/Thr221), were enhanced by RCN1 depletion, which was in turn neutralized by human osteoblast‐CM, even offset, indicating that RCN1 produced by osteoblasts facilitated proliferation and migration of NSCLC cells, in part, through suppressing ER stress, in which decreased GRP78, CHOP, IRE1α, p‐IRE1α, p‐PERK and p‐JNK were involved, consistent with other authors’ studies in liver cancer cells^38^ and prostate cancer cells.^40^ In addition, the data that the decreased PCNA and Bcl‐2 levels by RCN1 depletion were reversed by the addition of ER stress inhibitor 4‐PBA represented that RCN1 depletion‐induced ER stress‐triggered apoptosis, in agreement with previous studies.^38^, ^40^, ^41^ Therefore, we suggest that RCN1 suppressed ER stress by down‐regulating GRP78, CHOP, IRE1α, p‐IRE1α, p‐PERK and p‐JNK levels, promoting proliferation and migration of NSCLC cells.

Most lines of evidence demonstrate that canonical and non‐canonical ER stress can activate cytoprotective autophagy and contribute to tumour growth and therapy resistance.^33^, ^41^, ^42^ Herein, we found that RCN1 depletion induced autophagy in NCI‐H1299 cells, because shRCN1 vector result in increased Beclin1 and ATG5 levels, accompanied with decreased p‐mTOR and p‐ULK1 levels. It seemed that RCN1 depletion‐enhanced ER stress activate autophagy in NCI‐H1299 cells, consistent with those studies. However, further observation displayed that RCN1 depletion‐induced autophagy was not able to be suppressed by ER stress inhibitor 4‐PBA. On the contrary, the addition of 4‐PBA slightly elevated the Beclin1 and ATG5 levels in NCI‐H1299 cells transduced shRCN1 vector, implying that RCN1 depletion‐induced autophagy was not a downstream of RCN1 depletion‐induced ER stress in NCI‐H1299 cells. Some studies have demonstrated that the defective autophagy could promote ER stress.^43^, ^44^, ^45^ However, we found that an autophagy inhibitor 3‐MA reduced the CHOP level and expansion of ER, representing that the defective autophagy by 3‐MA suppressed ER stress. Li et al.’s study also indicates that 3‐MA inhibited UPR activation, thereby suppressing ER stress in mammalian cells.^46^ Y Tang and his colleagues have reported that the inhibition of autophagy attenuates ER stress‐evoked alveolar epithelial cells apoptosis in rat models of chronic obstructive pulmonary disease.^47^ Hence, we suggest that RCN1 depletion‐induced autophagy might be a positive regulator to enhance ER stress in NCI‐H1299 cells. Regarding the data that the expression levels of PCNA and Bcl‐2 were not markedly elevated in 3‐MA‐treated NCI‐H1299 cells transduced by shRCN1 vector, it is speculated that there might exist a complicated mechanism to regulate apoptosis, which was not completely dependent on RCN1 depletion‐induced ER stress or autophagy.

In conclusion, our findings determined that osteoblasts produced RCN1 to transfer into NSCLC cells partially through encapsulating in EVs. Furthermore, osteoblastic RCN1 contributed to proliferation and migration of NSCLC cells through suppressing ER stress that was associated with decreased GRP78, CHOP, IRE1α, p‐IRE1α, p‐PERK and p‐JNK, which could be positively regulated by self‐induced autophagy (Figure 5E). Therefore, RCN1 could be required for proliferation and migration of NSCLC cells regulated by osteoblast‐CM.

## CONFLICTS OF INTEREST

The authors confirm that there are no conflicts of interest.

## AUTHOR CONTRIBUTION

**Haijing Fu:** Data curation (lead); Formal analysis (lead); Investigation (lead); Methodology (lead); Supervision (supporting); Validation (supporting); Writing‐original draft (supporting). **Rui Chen:** Data curation (supporting); Investigation (supporting); Methodology (supporting); Software (supporting). **Yue Wang:** Data curation (supporting); Investigation (supporting); Methodology (supporting); Software (supporting). **Yang Xu:** Data curation (supporting); Investigation (supporting); Methodology (supporting). **Chun Xia:** Conceptualization (equal); Funding acquisition (lead); Project administration (equal); Resources (equal); Supervision (equal); Writing‐original draft (equal); Writing‐review & editing (equal). **bing zhang:** Conceptualization (equal); Project administration (equal); Resources (equal); Supervision (equal); Writing‐original draft (lead); Writing‐review & editing (equal).

## Supporting information

Figure S1

Figure S2

Figure S3

Figure S4

Supplementary Material

## Data Availability

The data support the findings of this study are available in the supplementary material of this article.
