# Supplementary figures and images for: Reticulocalbin 1 is required for proliferation and migration of non‐small cell lung cancer cells regulated by osteoblast‐conditioned medium
Source: J Cell Mol Med. 2021 Nov 7;25(24):11198–211. doi: 10.1111/jcmm.17040 (PMC8650041; doi:10.1111/jcmm.17040)

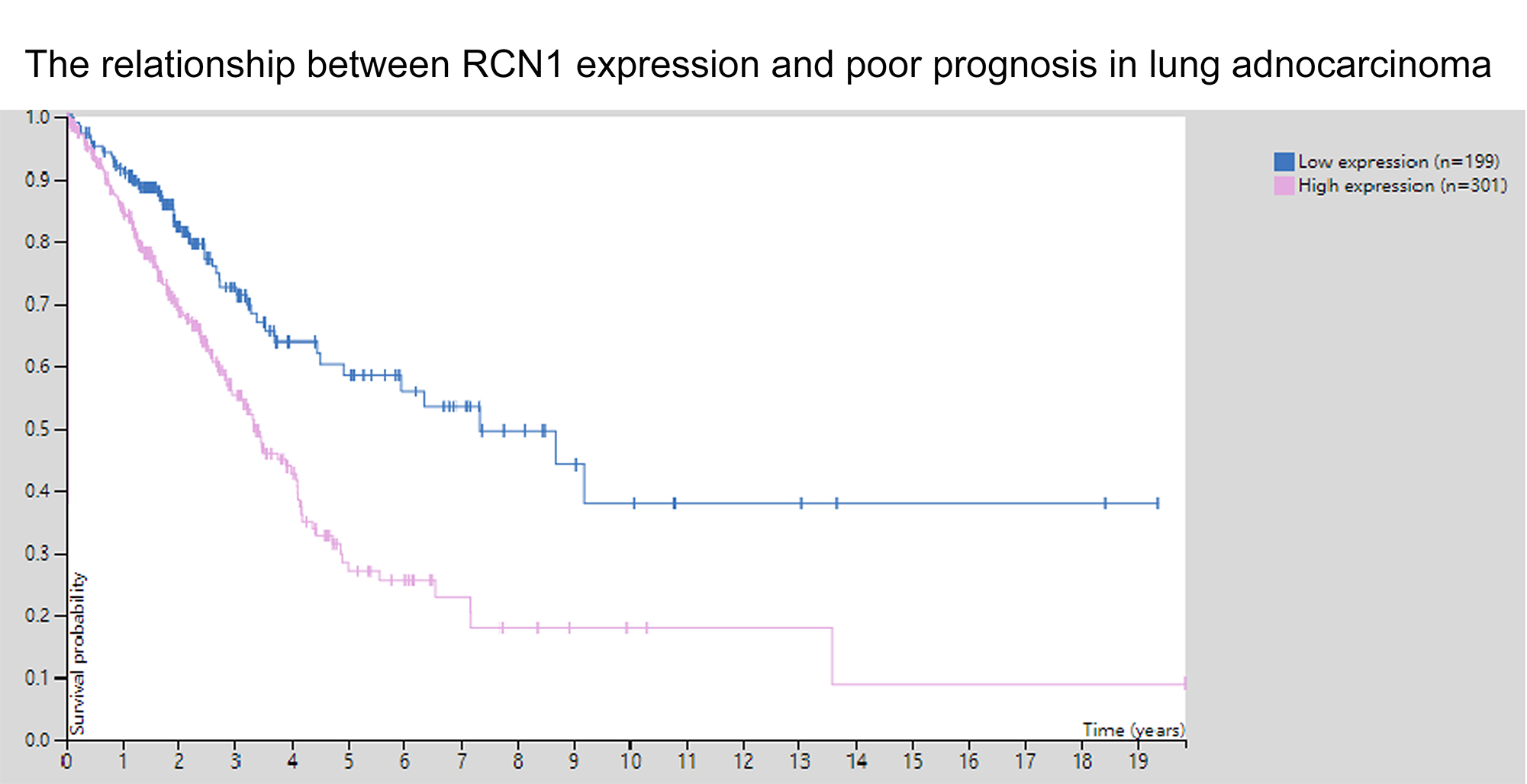

Supplement: Supplementary file 1 — Figure S1 [file JCMM-25-11198-s005.jpg]

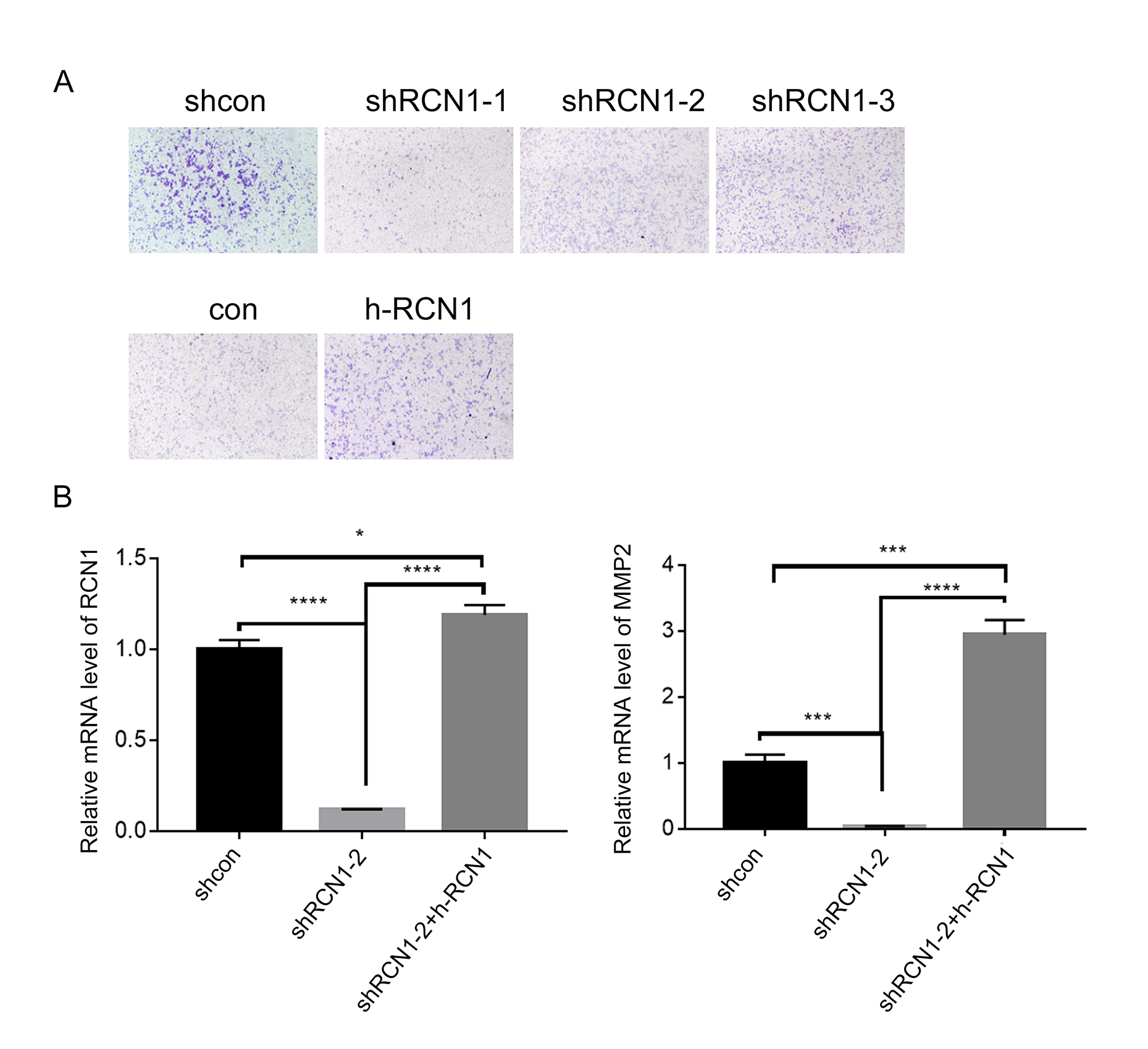

Supplement: Supplementary file 2 — Figure S2 [file JCMM-25-11198-s003.jpg]

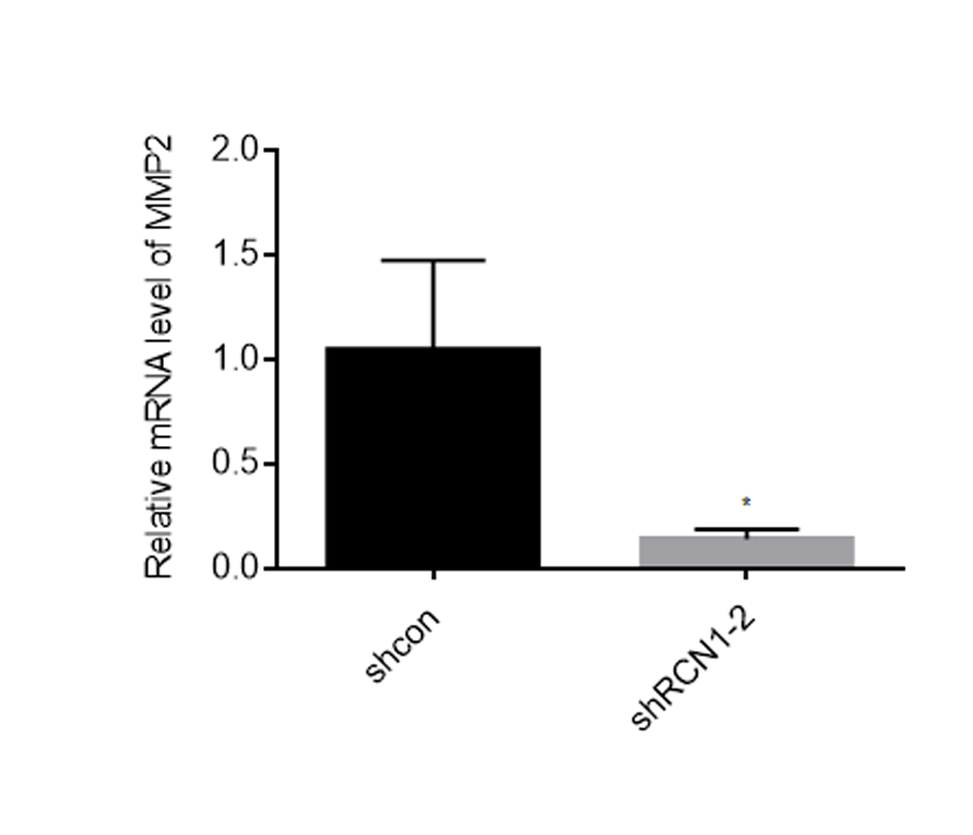

Supplement: Supplementary file 3 — Figure S3 [file JCMM-25-11198-s004.tif]

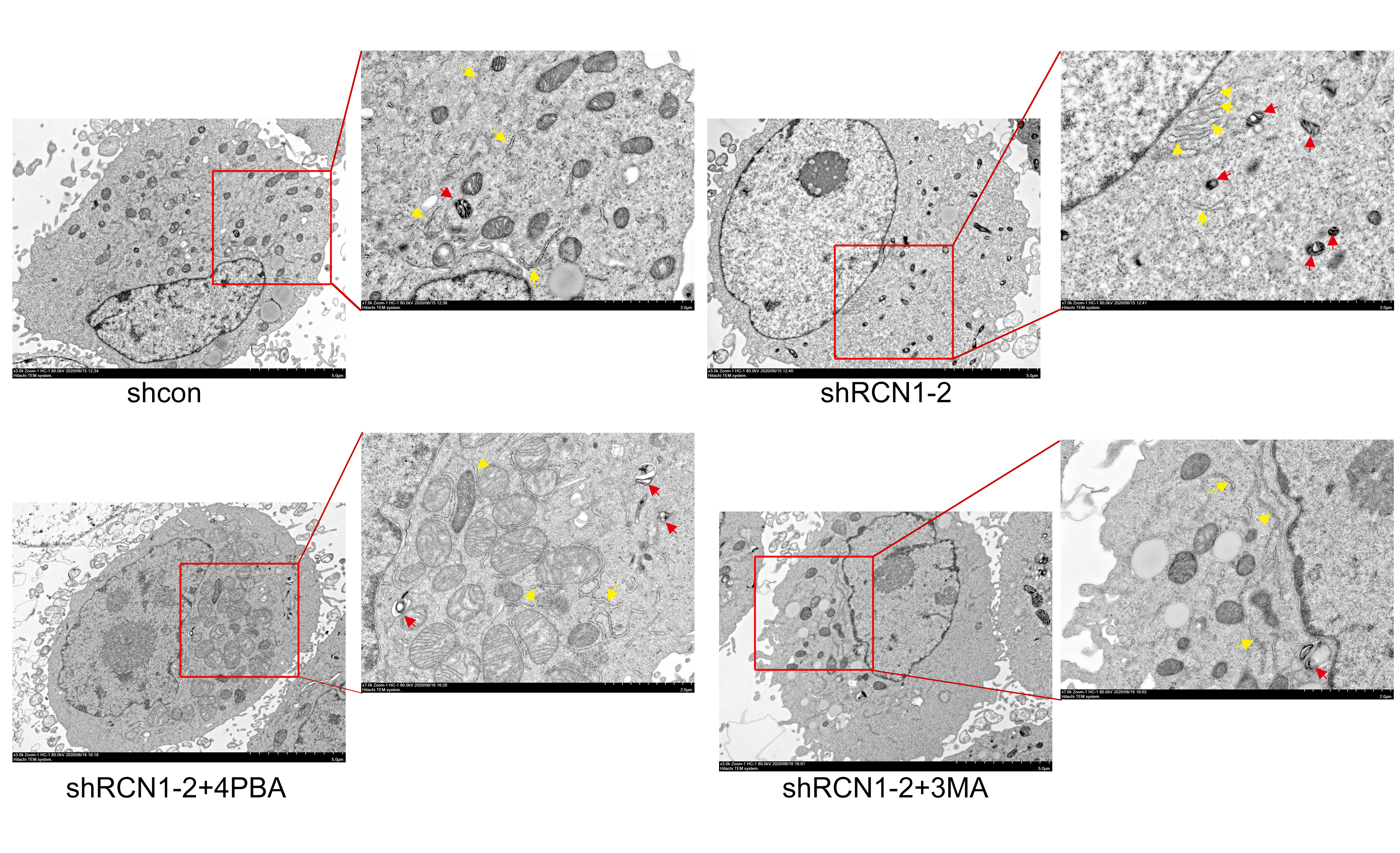

Supplement: Supplementary file 4 — Figure S4 [file JCMM-25-11198-s002.tif]
