# Supplementary material for: Reticulocalbin 1 is required for proliferation and migration of non‐small cell lung cancer cells regulated by osteoblast‐conditioned medium
Source: J Cell Mol Med. 2021 Nov 7;25(24):11198–211. doi: 10.1111/jcmm.17040 (PMC8650041; doi:10.1111/jcmm.17040)
Supplement: Supplementary file 5 — Supplementary Material [file JCMM-25-11198-s001.docx]

**Figure S1** Overexpressed RCN1 is correlated with poor prognosis and progression in lung cancer.

**Figure S2** The effect of shRCN1 vector on cell migration was rescued by h-RCN1 vector in NCI-H1299 cells. NCI-H1299 cells were transduced with lentiviral-mediated shRNA/RCN1-1/2/3 vectors or transfected with pHBLV-CMV-ZsGreen-RCN1 vector expressing RCN1. The cell migration was assessed using a wound healing assay as described in the Material and methods section (A). The mRNA level of MMP2 was detected using RT-PCR as described in the Material and methods section(B). The data are representative of three independent experiments (*p<0.05, ***p<0.001, ****p<0.0001).

**Figure S3** The effect of CM from rat osteoblasts transduced with shRNA/RCN1 vector on MMP2 mRNA expression in NCI-H1299 cells.NCI-H1299 cells were cultured in CM from rat osteoblasts transduced with shRNA/RCN1-2 vector. Relative mRNA level of MMP2 was measured via RT-PCR as described in the Material and methods section (*p<0.05).

**Figure S4** Observation of endoplasmic reticulum and autophagic vacuoles in NCI-H1299 cells using transmission electron microscopy. NCI-H1299 cells transduced with shRNA/RCN1-2 vector were treated with 4-PBA (1 mM) or 3MA (6 mM) for 48 h, and endoplasmic reticulum (indicated by yellow arrows) and autophagic vacuoles (indicated by red arrows) were observed under a transmission electron microscope.
